# The effects of different velogenic NDV infections on the chicken bursa of Fabricius

**DOI:** 10.1186/s12917-017-1071-y

**Published:** 2017-05-31

**Authors:** Y. W. Kristeen-Teo, S. K. Yeap, S. W. Tan, A. R. Omar, A. Ideris, S. G. Tan, N. B. Alitheen

**Affiliations:** 1 Institute of Bioscience, Universiti Putra Malaysia, 43400 Serdang, Selangor Malaysia; 2 Faculty of Veterinary Medicine, Universiti Putra Malaysia, 43400 Serdang, Selangor Malaysia; 3 Faculty of Biotechnology and Biomolecular Sciences, Universiti Putra Malaysia, 43400 Serdang, Selangor Malaysia

**Keywords:** Newcastle disease virus, B lymphocytes, Apoptosis, Oxidative stress

## Abstract

**Background:**

Virulent Newcastle disease virus (NDV) was reported to cause rapid depletion of chicken bursa of Fabricius. Severe pathological condition of the organ is commonly associated with high levels of virus replication, intense inflammatory response and also the degree of apoptosis. In this study, the responses of chicken bursa of Fabricius infected with two different strains of velogenic NDV, namely AF2240 and IBS002, were investigated by observing cell population changes, oxidative stress, viral replication and cytokine expression in the organ. Subsequently, apoptosis of enriched bursal IgM+ cells was determined to help us elucidate possible host pathogen relationships between the chicken bursa of Fabricius and NDV infection.

**Results:**

The depletion of IgM+ cells and infiltration of macrophages were observed to be higher in bursa infected with AF2240 as compared to IBS002. In line with the increment of the macrophage population, higher nitric oxide (NO) and malondialdehyde (MDA) contents which indicated higher oxidative stress were also detected in bursa infected with NDV AF2240. In addition, higher pro-inflammatory cytokines and chemokine gene expression such as chicken CXCLi2, IL-18 and IFN-γ were observed in AF2240 infected bursa. Depletion of IgM+ cells was further confirmed with increased cell death and apoptosis of the cells in AF2240 infected bursa as compared to IBS002. However, it was found that the viral load for NDV strain IBS002 was comparatively higher than AF2240 although the magnitude of the pro- inflammatory cytokines expression and cell apoptosis was lower than AF2240.

**Conclusion:**

The results of our study demonstrated that infection of NDV strains AF2240 and IBS002 caused apoptosis in bursa IgM+ cells and its severity was associated with increased expression of pro-inflammatory cytokines/chemokine, macrophage infiltration and oxidative stress as the infection duration was prolonged. However, of the two viruses, we observed that NDV AF2240 induced a greater magnitude of apoptosis in chicken bursa IgM+ cells in comparison to IBS002. This might be due to the high level of oxidative stress and inflammatory cytokines/chemokine as well as lower IL10 expression which subsequently led to a high rate of apoptosis in the chicken bursa of Fabricius although the detected viral load of AF2240 was lower than IBS002.

**Electronic supplementary material:**

The online version of this article (doi:10.1186/s12917-017-1071-y) contains supplementary material, which is available to authorized users.

## Background

Newcastle disease virus is classified under the order Mononegavirales in the family Paramyxoviridae, which is divided into two subfamilies, Paramyxovirinae and Pneumovirinae [1]. It was reported that a few of the first outbreaks of Newcastle disease occurred in Newcastle Upon Tyne, England and also Java, Indonesia during the early 1900s [2]. The outbreak in Southeast Asia during 1926 spread to most of the other regions of the world and this outbreak was thought to be caused by three different genotypes of NDV which were types II, III and IV [3]. However, a similar pattern of outbreaks was also observed earlier in some areas of Central Europe [4]. In the late 1970s, NDV genotypes V and VI caused a panzootic outbreak in the Middle East [5]. In the early 2000s, NDV outbreaks in Asia were recorded in Taiwan and China caused by NDV genotype VII [6]. This virus was shown to survive when airborne in small particles, either in the laboratory or open air [7]. When infected, birds may show variable symptoms such as loss of appetite, weight loss, weakness, diarrhea and nervous signs [2, 8]. Post mortem examination showed severe hemorrhagic lesions of the intestine and proventriculus [9]. The pathological manifestations observed upon NDV infections are contributed by factors such as the strain of the virus, species of birds, concurrent diseases and preexisting immunity which can affect the severity of the disease [10].

Avian bursa of Fabricius had been identified to play an important role in the development of B lymphocytes as studies showed that the surgical removal of this organ during the embryonic stage of a bird compromised its antibody production thus leading to immunosuppresion [11, 12]. The organ serves as an essential site for generation of antibody diversity by gene conversion. It was reported to serve as a primary lymphoid organ until the age of five to 6 weeks and after that as a peripheral lymphoid organ [13]. Primarily this organ consisted of 98% B lymphocytes [14] but infiltration of other cells, such as T cells and macrophages was observed in response to viral infection [15]. The bursa of Fabricius was involved in host responses against poultry virus not only by Infectious bursal disease virus (IBDV) [16–18], in which the virus infection caused cell population and cytokine expression changes but also upon NDV infection. Several reports showed that virulent NDV strains cause rapid depletion of the bursa of Fabricius [19–21] and the severe pathology in immune organs caused by virulent NDV strains is associated with high levels of virus replication and an intense inflammatory response [22, 23]. Also, apoptosis was reported to play a role in the pathogenesis of NDV as well. It was demonstrated that the amount of apoptosis was proportional to the severity of the clinical disease elicited by the strains [20, 24]. In this study, we investigated the response of chicken bursa of Fabricius upon infection with two different strains of Malaysian isolates of velogenic NDV. Oxidative stress caused by the virus infection was also evaluated by detecting the nitric oxide content in the organ. Apoptosis studies on B lymphocytes, which are the largest cell population in the organ, was done and correlated with viral load detection in the organ to help us elucidate possible host pathogen relationships between the chicken bursa of Fabricius and Newcastle disease virus.

## Methods

### Experimental design

The study was approved by the Institutional Animal Care and Use Committee (IACUC), Universiti Putra Malaysia (UPM). SPF layer chicken eggs (*n* = 80) were obtained from the Veterinary Research Institute (Ipoh, Malaysia), hatched in the Laboratory of Vaccines and Immunotherapeutics, Institute of Biosciences, UPM and raised until the age of 35 days old. The trial was conducted in UPM Faculty of Veterinary Medicine experimental animal facility where the different groups of birds were kept separately in different rooms and cages. The birds were then randomly divided into three groups with the first two groups (*n* = 30) being infected with 10^6^ ELD50 of NDV strain AF2240 and IBS002, respectively. The viruses were delivered to the chickens via ocular (50 μl) and nasal (50 μl) routes using 1 ml syringe. The remaining birds were treated with PBS and served as the control group. Ten birds from each infected group were then observed, sacrificed and necropsized at days 1, 3 and 4 post infection whereas the control chickens were sacrificed at day 4. Chicken bursa of Fabricius was removed and cut into two parts, which one for oxidative stress study and RNA extraction for gene expression purposes. Meanwhile the other part of the organ was used for enrichment of IgM+ cells, which were subsequently subjected to apoptosis related assays such as acridine orange/propidium iodide (AO/PI) double staining, Annexin V apoptosis and DNA cell cycle assays. Prior to cell isolation, a cell count was performed using a hemacytometer and a portion of the cells (2 × 10^6^ cells) was subjected to flow cytometry analysis to for cell population study.

### Virus isolates

The viruses used in this study are velogenic isolates Chicken/Malaysia/Perak/AF2240/1960 (AF2240) and Commercial broiler chicken/Malaysia/Johor/IBS002/2011 (IBS002). NDV AF2240 is a viscerotropic velogenic, genotype VIII strain that is used as a vaccine challenge virus in Malaysia [25]. The intracerebral pathogenicity index (ICPI) for AF2240 was 1.9 [26] while its mean death-time (MDT) was 48 h [27]. On the other hand, NDV isolate IBS002 was characterized as a virulent genotype VII strain based on the multiple basic amino acid motif of the fusion (F) cleavage site 112RRRKGF117 and length of the C-terminus extension of the hemagglutinin-neuraminidase (HN) gene with the ICPI and MDT at 1.76 and 51.2 h respectively [28]. Both viruses are belonged to and owned by Institute of Bioscience, Universiti Putra Malaysia.

### Immunophenotyping of SPF chicken bursa of Fabricius

To study the cell population changes in chicken bursa when infected with NDV AF2240 and IBS002, immunophenotyping using flowcytometry was conducted. Single cells were isolated from chicken bursa of Fabricius at days 1, 3 and 4 post infection. In brief, the organs were removed (*n* = 5), washed with PBS and cut into pieces using a scalpel, before being meshed through 70 μm sterile wire mesh screen (SPL Life Sciences, China). The single cells were then collected and washed twice in PBS-BSA-EDTA. After that, the pellet was subjected to Histopaque 1119 (Sigma, USA) at 400 g for 40 min. The lymphocyte layers were collected and washed twice again with PBS-BSA-EDTA. A cell count was performed using a hemacytometer and a number of 1 × 10^6^ cells/tube were incubated with 10 μg/10 μL mouse anti-chicken CD3-FitC, clone CT-3 and mouse anti-chicken CD4-PE, clone CT-4 (tube 1), mouse anti-chicken CD3-FitC, clone CT-3 and mouse anti-chicken CD8α-PerCp, clone CT8 (tube 2), mouse anti-chicken IgM-PE, clone M-1 (tube 3) and mouse anti-chicken monocyte/macrophage-PE, clone KUL01 (tube 4) antibodies (Southern Biotech, USA) for 45 min in the dark. The cells were then washed again with PBS and lastly resuspended in 1 ml PBS. Flow cytometer analysis was carried out using a FACSCalibur machine with CellQuest Pro software (BD Bioscience, USA). A total of 20,000 events were analyzed via gating the chicken lymphocytes using forward and side scatter before the data for positive staining of different fluorescence were collected. The average cell numbers of CD4, CD8, IgM and macrophages are listed in Table 1.

**Table 1:** Average cell number of different lymphocytes subsets after infected with NDV AF2240 and NDV IBS002

Groups		Average cell numbers			
		Control	Day 1	Day 3	Day 4
CD4	NDV AF2240	0.79 × 10^5^ ± 50	0.34 × 10^5^ ± 12^*^	0.46 × 10^5^ ± 21^**^	1.46 × 10^5^ ± 52^***a^
	NDV IBS002	0.79 × 10^5^ ± 50	0.33 × 10^5^ ± 22^*^	0.46 × 10^5^ ± 12^**^	1.08 × 10^5^ ± 20^***b^
CD8	NDV AF2240	0.27 × 10^5^ ± 12	0.71 × 10^5^ ± 22^*a^	1.10 × 10^5^ ± 115^**a^	1.40 × 10^5^ ± 21^***a^
	NDV IBS002	0.27 × 10^5^ ± 12	0.23 × 10^5^ ± 10^*b^	0.65 × 10^5^ ± 13^**b^	1.58 × 10^5^ ± 11^***b^
IgM	NDV AF2240	1.78 × 10^6^ ± 36	1.76 × 10^6^ ± 83	1.43 × 10^6^ ± 29^*a^	1.24 × 10^6^ ± 101^**a^
	NDV IBS002	1.78 × 10^6^ ± 36	1.73 × 10^6^ ± 53	1.64 × 10^6^ ± 24^*b^	1.37 × 10^6^ ± 115^**b^
Macrophage	NDV AF2240	0.69 × 10^5^ ± 21	0.35 × 10^5^ ± 24^*^	0.95 × 10^5^ ± 47^**a^	2.86 × 10^5^ ± 16^***a^
	NDV IBS002	0.69 × 10^5^ ± 21	0.40 × 10^5^ ± 66^*^	1.08 × 10^5^ ± 63^**b^	1.70 × 10^5^ ± 15^***b^

Data representing average cell number of CD4+, CD8+, IgM and macrophages with standard deviation from chicken bursa of Fabricius cells of five chickens from each group.

Different asterisk signs show significant difference between infection time points (*P* ≤ 0.05).

Different alphabets show significant difference between different strains of NDV (*P* ≤ 0.05).

### Oxidative stress (NO and MDA detection)

Upon NDV AF2240 and IBS002 infection, oxidative stress in chicken bursa of Fabricius was observed and compared by measuring nitric oxide (NO) and malondialdehyde (MDA) level. Chicken bursas of Fabricius at different time point after being infected with NDV AF2240 and IBS002 were cut and meshed before the organ homogenate was subjected to NO and MDA detection (*n* = 5 from each group). For NO evaluation, 150 μL of the sample homogenate was added with 20 μL of Griess Reagent (Invitrogen, USA) and 130 μL of deionized water in a microplate. The mixture was incubated for 30 min at room temperature. After the incubation, the sample absorbance was measured at 548 nm using μQuant ELISA Reader (Bio-Tek Instruments, USA). Meanwhile, MDA activity was determined by measuring the thiobarbituric acid-reactive substance (TBARS). In brief, 100 μL of bursa homogenate was added with 12.5 μL butyhydroxytoulene (8.8 mg/mL), 250 μL 30% trichloroacetic acid and 400 μL of PBS before the mixture was mixed and incubated on ice for 2 h. Subsequently, the mixture was spun at 300 g for 15 min and the supernatant was collected and added with 37.5 μL of 0.1 M EDTA and 125 μL 1% thiobarbituric acid before being subjected to boiling for 15 min. The mixture would then turn pink and the absorbance was read at 532 and 600 nm using a μQuant ELISA Reader (Bio-Tek Instruments, USA). The absorbance measured was then compared with the standard made with malonaldehyde tetramethylacetal solutions at different concentrations and the MDA activity was expressed as nmol MDA/g protein.

### Detection of viral load and cytokine expression by real time PCR

TaqMan probe based real time PCR was used to determine viral load in chicken samples infected with NDV AF2240 and IBS002. RNA was isolated from both crude cells and enriched IgM+ cells. Bursas of Fabricius from the control and infected chickens were removed (*n* = 5 from each group) and RNA from each sample was extracted using TRIZOL (Invitrogen, USA) following the instruction recommended by the manufacturer. The concentration and purity of the extracted RNA were measured by using a spectrophotometer (DU®730, Beckman Coulter, USA) at the wavelength of 260 nm over 280 nm before being subjected to cDNA synthesis using iScriptcDNA Kit (Biorad, USA) incubated in a thermal cycler for 5 min at 25 °C, 30 min at 42 °C and 5 min at 85 °C. The cDNA was then subjected to NDV viral load detection using usingiQSupermix (Biorad, USA) together with forward primer 5′-TCCGCAAGATCCAAGGGTCT-3′, reverse primer 5′-CGCTGTTGCAACCCCAAG-3′ and Taqman probe 5′-(FAM)-AAGCGTTTCTGTCTCCTTCCTCCA-(BHQ)-3′. The cycling program consists of 95 °C for 5 min and followed by 39 cycles of 95 °C for 10 s, 56 °C for 30 s and 72 °C for 20 s. Standard curves for detection of NDV AF2240 and IBS002 was provided in Additional file 1: Figure S1. As for cytokines expression, following primers and probes were used: forward primer 5′-GTGAAGAAGGTGAAAGATATCATGGA-3′, reverse primer 5′- GCTTTGCGCTGGATTCTCA-3′ and probe 5′-(FAM)-TGGCCAAGCTCCCGATGAACGA-(BHQ1)-3′ for IFN- ɣ expression; forward primer 5′-GCCCTCCTCCTGGTTTCAG-3′, reverse primer 5′-TGGCACCGCAGCTCATT -3′ and probe 5′-(FAM)-TCTTTACCAGCGTCCTACCTTGCGACA-(BHQ1)-3′ for CXCLi2 expression; forward primer 5′-AGGTGAAATCTGGCAGTGGAAT-3′, reverse primer 5′-ACCTGGACGCTGAATGCAA-3′ and probe 5′-(FAM)-CCGCGCCTTCAGCAGGGATG-(BHQ1)-3′ for IL18 expression; forward primer 5′-CATGCTGCTGGGCCTGAA-3′, reverse primer 5′-CGTCTCCTTGATCTGCTTGATG-3′ and probe 5′-(FAM)-CGACGATTCGGCGCTGTCACC-(BHQ1)-3′ for IL10 expression; as well as forward primer 5′-GAACGGGAAACTTGTGAT-3′, reverse primer 5′-GACTCCACAACATACTCA-3′ and probe 5′-(FAM)-CGCCATCACTATCTTCCAGG-(BHQ1)-3′ for GAPDH expression as reference gene. The real time PCR cycling program was set up as 1 cycle of 95 °C for 5 min, followed by 40 cycles of 95 °C for 20 s, 58 °C for 20 s (IFN- ɣ, CXCLi2, IL18 and IL10) or 60 °C for 30 s (GAPDH), depending on primer annealing temperature. Viral load was calculated by using standard curve (Additional file 1: Figure S1) with the following formula:

$$ Log\  Quantity=\frac{Ct- b}{m} $$

Ct = cycle threshold value.

b = y-intercept.

m = slope.

Relative fold change for CXCLi2, IFN- ɣ and IL18 was calculated using 2^-Δ∆Ct^ method. Meanwhile, IL10 was expressed as relative ΔCt gene expression normalized with GAPDH because no IL10 was detected on day 1 post infection and uninfected controls.

### Isolation of enriched B lymphocyte population and cell viability assessment

At days 0, 1, 3 and 4 post infection, single cells were harvested from chicken bursa of Fabricius of SPF chickens (*n* = 10 from each group). The cells were then stained with 10 μg/10 μL PE-labelled IgM for 45 min and washed twice with PBS. Subsequent to that, the cells were labelled with 20 μL of anti-PE microbeads for 30 min prior to magnetic isolation of pure B (IgM^+^) cell population using a LS column (Miltenyi Biotec, Germany). The enriched B cells were then washed twice with PBS before proceeding to cell viability testing by using the colorimetric 3-(4,5-dimethylthiazol-2-yl)-2,5-diphenyl tetrazolium bromide (MTT) assay as described previously [29].

### Acridine orange/propidium iodide (AO/PI) double staining assay

**Fig. 1:**
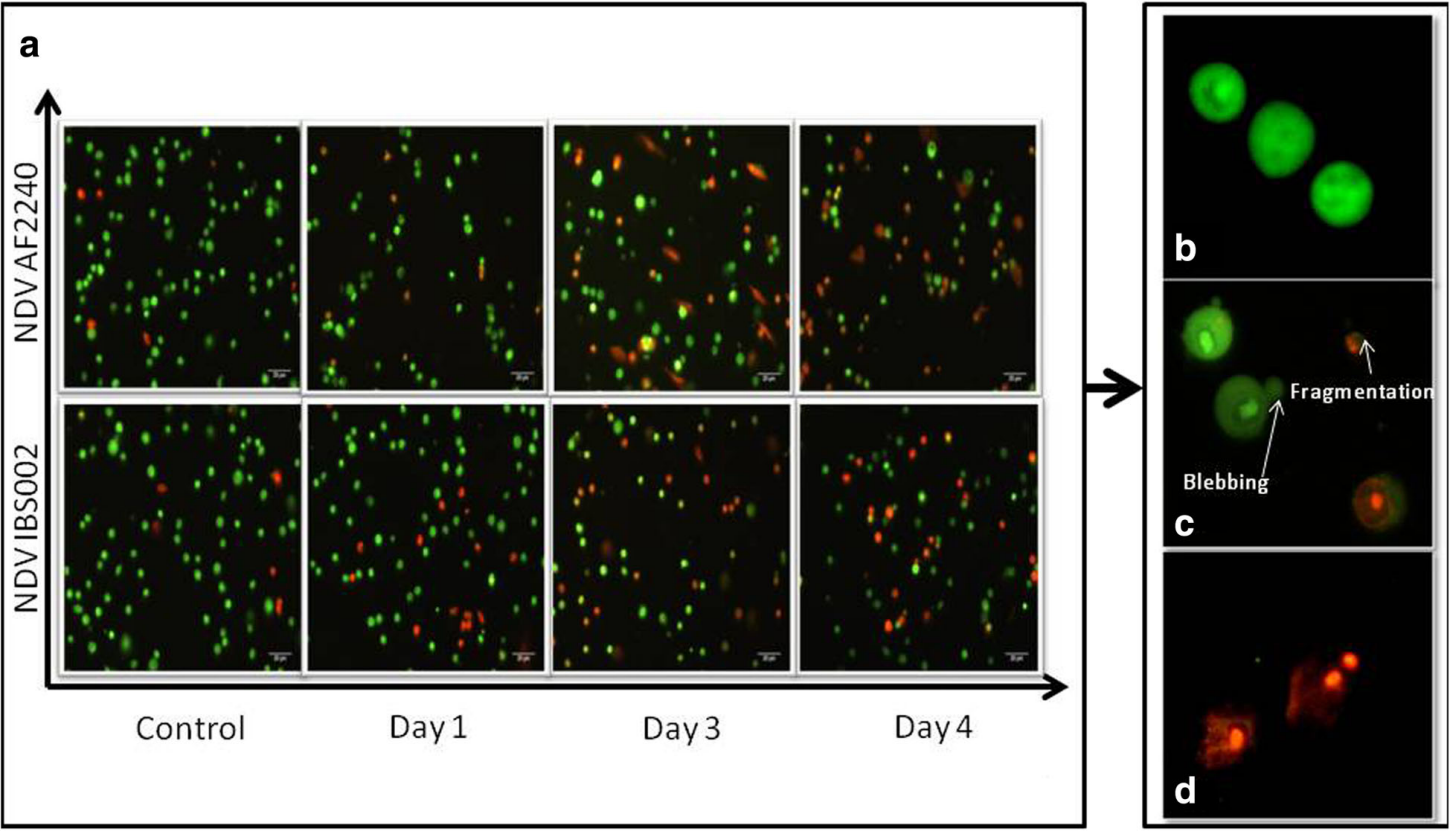
AO/PI assay of chicken enriched B lymphocytes infected with (**a**) NDV AF2240 and NDV IBS002 at day 1, 3 and 4 post infections, magnification 400×; **b** Viable cells; **c** Early Apoptotic cells where *arrow* indicating membrane blebbing and chromation condensation, red cells are late apoptotic cells; **d** Late apoptotic or necrotic cells, magnification 400×; AO (excitation: 488 nm, emission: 545 nm), PI (excitation: 535 nm, emission: 617 nm)

The enriched IgM + B cell suspension was washed with PBS twice and 10 μL of the cells were put on a glass slide. Acridine orange (50 μg/ml) and propidium iodide (50 μg/ml) were mixed together at a 1:1 ratio to a total volume of 1 ml. After that, 10 μl of the mixture was added to the cells on a glass slide and resuspended carefully. The slide was then analyzed using a fluorescent microscope (Nikon, Japan) within 30 min. This assay permitted clear differentiation of viable and nonviable cells using two different fluorescent dyes. Through counting a total population of 200 cells, the percentage of viable, apoptotic and necrotic cells were determined. Besides, cells with condensed chromatin and blebbing were also considered as apoptotic cells (Fig. 1). The experiment was repeated using three biological replicates for each group.

### Annexin V apoptosis assay

Enriched IgM + B lymphocytes of every group were harvested and subjected to Annexin V apoptosis assay. The cells were washed with PBS and stained with Annexin V FITC (BD Biosciences, USA) for 20 min prior to analysis using a FACSCalibur machine with CellQuest Pro Software (BD Bioscience, USA). The assay was conducted in triplicates.

### DNA cell cycle analysis

A total of 1 × 10^6^ enriched IgM+ B cells was collected from each sample group and fixed in 80% ethanol overnight at a temperature of −20 °C. The samples were then re-pelleted and washed twice by using PBS-Sodium-Azide-EDTA buffer. Finally, the pellet was dissolved in 1 mL of PBS buffer containing 0.1% Triton X-100, 10 mM EDTA, 50 μg/mL RNase and 2 μg/ml SYTOX green in the dark, followed by incubation for at least 30 min on ice. The samples were then subjected to analysis by flow cytometry using a FACSCalibur machine with CellQuest Pro software (BD Biosciences, USA).

### Statistical analysis

The Graphpad Prism 6.0 software was used to aid the statistical analysis of the results. The results were expressed as mean ± standard deviation and subjected to one way ANOVA or two-way ANOVA test, followed by the Bonferroni procedure with infection time points and virus genotypes as main effects. Results with *P* < 0.05 were considered as being statistically significant.

## Results

### Immunophenotyping

In chicken bursa, cell population changes were observed when the animals were infected with different strains of velogenic NDV. The average numbers of different lymphocyte subsets were counted and the results are shown in Table 1. Similar patterns of CD4+ cell population changes were observed upon infection by both NDV AF2240 and IBS002. The infection caused depletion of CD4+ T cells on days 1 and 3 post infection, but on the fourth day, NDV AF2240 caused the CD4+ cell number to increase to 1.46 × 10^5^ from the initial 0.79 × 10^5^ cells while NDV IBS002 increased the number to 1.08 × 10^5^ cells. CD8+ cells were found to gradually increase along with the time of infection by both viruses, with higher increments during day 1 and day 3 post infection caused by NDV AF2240 infection, whereas on the fourth day of infection, higher CD8+ cell number was observed in the chicken bursa infection by NDV IBS002. B lymphocytes, which were measured by the expression of IgM, were reported to decline significantly in cell number upon infection with both genotypes of NDV starting on day three after infection. However, infection with NDV AF2240 resulted in a greater decline in IgM+ cell number from an initial figure of 1.78 × 10^6^ ± 36 cells to 1.24 × 10^6^ ± 101 cells (Table 1) compared to NDV IBS002 for which the cell number of IgM+ cells reached 1.37 × 10^6^ ± 115 cells at day four post infection (Table 1). Also, it was observed that the macrophage population increased upon infection with both genotypes of NDV but NDV AF2240 resulted in a higher peak in macrophage cell number at 2.86 × 10^5^ ± 16 cells compared to NDV IBS002 at 1.70 × 10^5^ ± 15 cells on the fourth day after infection.

### Oxidative stress evaluation (NO and MDA detection)

**Fig. 2:**
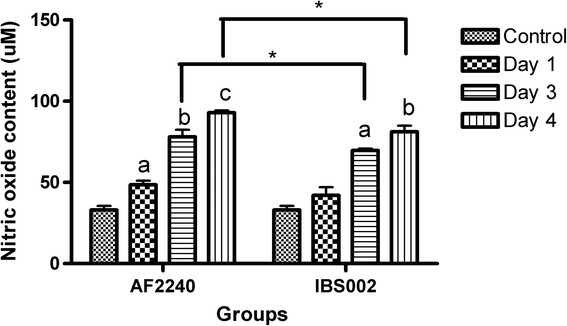
The nitric oxide content in the bursa of 3-week-old chicken infected with NDV AF2240 and IBS002 at day 1, 3 and 4 post infection. Significant differences were observed using two-ways ANOVA by using strains of NDV and infection time points as effectors. *Asterisk signs* show significant difference between different strains of NDV (*P* ≤ 0.05) while different *alphabets* show significant difference between different infection time points (*P* ≤ 0.05)

**Fig. 3:**
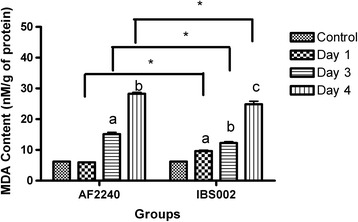
The MDA content in the bursa of 3-week-old chicken infected with NDV AF2240 and IBS002 at day 1, 3 and 4 post infection. Significant differences were observed using two-ways ANOVA by using strains of NDV and infection time points as effectors. Asterisk signs show significant difference between different strains of NDV (*P* ≤ 0.05) while different alphabets show significant difference between different infection time points (*P* ≤ 0.05)

Upon infection with virus, NO is considered as a pro-inflammatory mediator due to its over production. The level of NO in the chicken bursa of Fabricius was measured at 1, 3 and 4 days post infection. Figure 2 shows the NO levels detected in the chickens’ bursa after infection with the two viral genotypes respectively. According to Fig. 2, the level of nitric oxide content in chicken bursa of Fabricius upon NDV AF2240 infection increased from an initial of 33.06 ± 4.2 μM of nitric oxide to the highest level of 92.89 ± 2.5 μM on the fourth day of infection whereas NDV IBS002 caused increment of nitric oxide level to 81.02 ± 6.4 μM in the organ. A similar trend of increment in MDA level was also observed in the chicken bursa of Fabricius after infection with the two different genotypes of NDV. Figure 3 shows that NDVAF2240 caused a significant elevation of MDA content in chicken bursa starting on day 3 post infection compared to the control group and reached its highest peak during day 4 at 28.26 ± 1.6 nM of MDA/ g of protein. Meanwhile, IBS002 caused a significant increase of the MDA level at day 1 post infection and reached 24.84 ± 0.5 nM of MDA/g of protein at day 4.

### Detection of viral load and cytokines by real time PCR

**Fig. 4:**
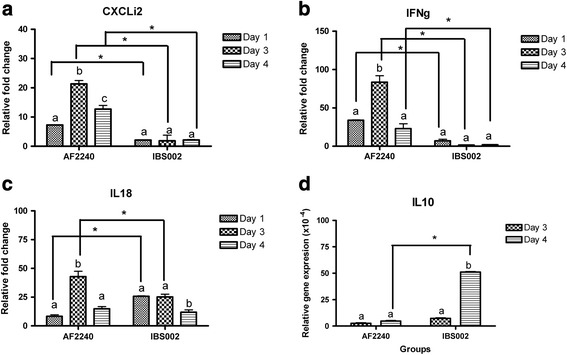
The normalized fold expression of (**a**) CXCLi2, (**b**) IFN-ɣ and (**c**) IL-18 and normalized Cq value of (**d**) IL10 in the bursa of 3-week-old chickens infected with NDV AF2240 or IBS002 at 1, 3 and 4 day post infection. Significant differences were observed using two-ways ANOVA by using strains of NDV and infection time points as effectors. *Asterisk signs* show significant difference between different strains of NDV (*P* ≤ 0.05) while different *alphabets* show significant difference between different infection time points (*P* ≤ 0.05)

The results showed that both viruses RNA could be detected in the samples starting on day 3 post infection and that the viral load increased on the following day. Table 2 shows that a higher viral load was detected in both chicken bursa and IgM + cells infected by NDV IBS002 compared to AF2240. On the other hand, Fig. 4 shows the expressions of IFN-ɣ, CXCLi2 and IL-18 detected in chicken bursa upon infection. As is shown in Fig. 4, the infection of NDV AF2240 significantly increased expression of IFN-ɣ starting on day 1 post infection as compared to the control group and then this was further elevated to 83 – fold change on day 3. On day 4 post infection, expression of IFN-ɣ was recorded at 22-fold compared to the control group. Both IL-18 and CXCLi2 gene expressions were also up-regulated during the infection of AF2240. The IL-18 expressions were up-regulated at 8-, 42- and 14 - folds at days 1, 3 and 4 post infection while the CXCLi2 expressions at 7-, 21- and 12- folds (Fig. 4). Significant changes of IFN-ɣ, IL-18 and CXCLi2 gene expressions were observed with NDV AF2240 infection at all different time points of infection. Meanwhile, no significant changes of IFN-ɣ and CXCLi2 were observed in chicken bursa upon NDV IBS002 infection. The expression of IL-18 was increased to 25 – fold at days 1 and 3 post infection, but decreased to 11-fold on the next day. Conversely, IL-10 was detected on day 3 and day 4 post infection of NDV AF2240 and IBS002 in the bursa (Fig. 4) but none on day 1 and uninfected control.

**Table 2:** TaqMan real time PCR result of detection of NDV AF2240 and IBS002 virus in the chicken bursa and IgM^+^ cells population isolated from chicken bursa of Fabricius

	Samples	C (t) mean		Viral copy number (log 10)	
		AF2240	IBS002	AF2240	IBS002
	Control	N/D	N/D	N/D	N/D
Bursa of Fabricius	Day 1	N/D	N/D	N/D	N/D
	Day 3	29.85 ± 0.28	26.24 ± 0.88	10.34^*a^	11.43^*b^
	Day 4	28.33 ± 0.18	23.20 ± 0.53	10.80^*a^	12.34^**b^
IgM + cells	Control	N/D	N/D	N/D	N/D
	Day 1	N/D	N/D	N/D	N/D
	Day 3	35.10 ± 0.27	28.74 ± 0.03	8.76^*a^	10.68^*b^
	Day 4	28.48 ± 0.16	26.82 ± 1.3	10.10^**a^	11.25^**b^

N/D stands for not detectable. No amplification of NDV gene was detected throughout whole qPCR cycling program.

All results represent the mean ± standard deviation

Different asterisk signs show significant different between infection time points (*P* ≤ 0.05).

Different alphabets show significant different between different strains of NDV (*P* ≤ 0.05).

NDV AF2240, R2 = 0.984, Slope = 3.13

### Cell viability and apoptosis assay

**Fig. 5:**
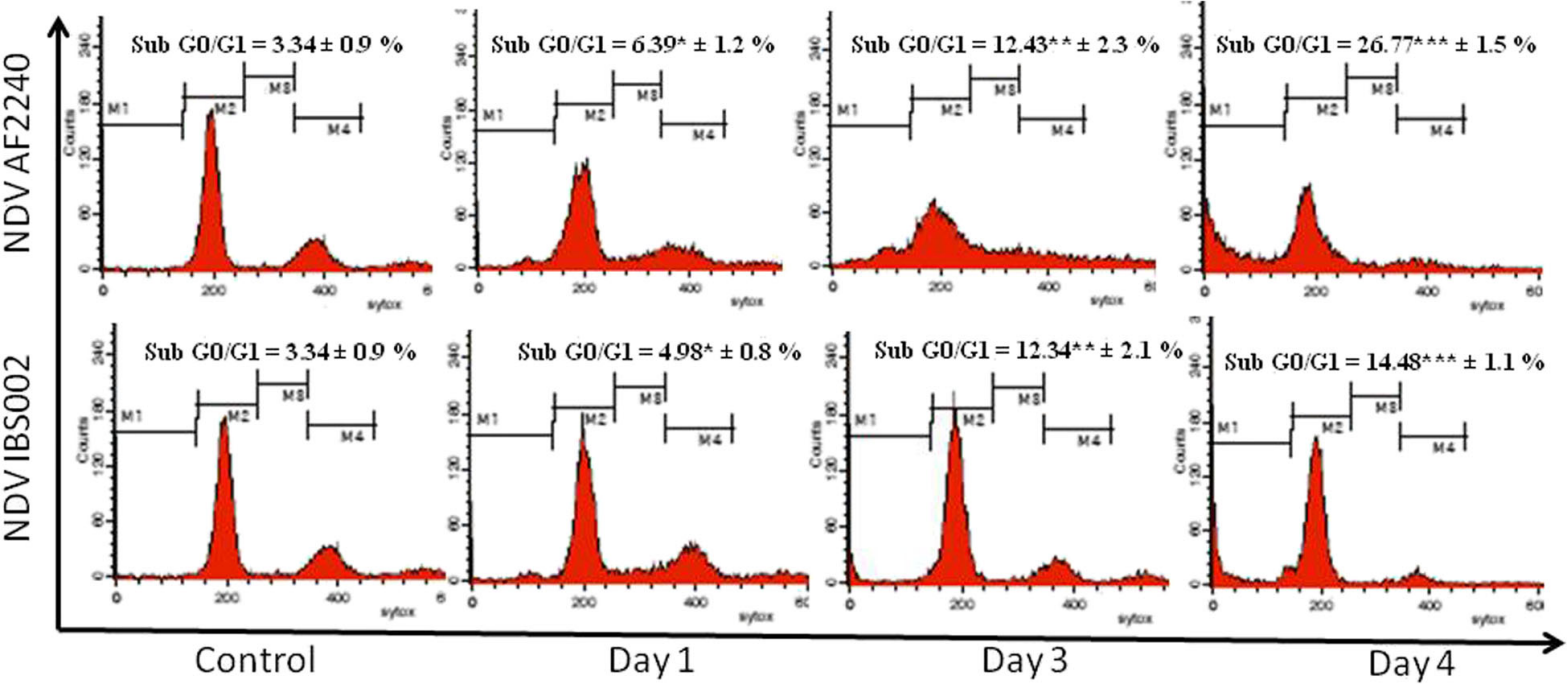
Pertubations of cell cycle phases of B lymphocytes in the bursa of chicken infected with NDV AF2240 and NDV IBS002 at day 1, 3 and 4 post infections. The sub G0/G1 population differences between the control group and treated group were determined by one-way ANOVA. Groups labelled with different superscript are significantly different (*P* ≤ 0.05)

The cell viability of enriched B lymphocytes, which are the largest cell population in chicken bursa of Fabricius was examined upon infection with the NDV viruses (Table 3). The results showed that the infection of NDV virus caused increment of cell death events in IgM cells in chicken bursa of Fabricius as the duration of infection increased (Table 3). MTT assay was performed and the percentages of viable cells of virus infected groups were normalized with the control group. It was found that NDV AF2240 caused higher cell death in the enriched B lymphocytes compared to NDV IBS002 (Table 3). These results were further confirmed with the apoptosis assays using AO/PI and Annexin V binding assays. As is shown in Table 4, it was found that infection of the chicken bursa by NDV AF2240 caused a higher percentage of apoptotic IgM+ cells in the organ. A similar trend of results was observed in the Annexin V binding assay where a greater number of Annexin V positive IgM+ cells were detected in chicken bursa during infection with NDV AF2240 (Table 3). In agreement with these results, it was observed that the infection of NDV viruses caused increment of the sub G0/G1 population in the enriched IgM+ cells. DNA fragmentation, which is one of the apoptosis hallmarks, can be indicated by the occurrence of the sub G0/G1 population. As is shown in Fig. 5, the population of sub G0/G1 increased over the infection time of NDV AF2240 and IBS002. It was also shown that as in the previous findings, NDV AF2240 caused higher frequency of DNA fragmentation compared to IBS002.

**Table 3:** MTT assay, AO/PI apoptotic population and Annexin V apoptotic population of chicken bursa of Fabricius enriched IgM^+^ B lymphocytes infected with NDV AF2240 and IBS002. The differences between the control group and infected group were determined by one-way ANOVA

Assays	NDV strains	Control	1 d.p.i	3 d.p.i	4 d.p.i
MTT viable IgM cells (%)	AF2240	100 ± 0.32	81.48 ± 0.36^*^	60.86 ± 0.44^**^	42.06 ± 0.19^***a^
	IBS002	100 ± 0.32	85.42 ± 0.22^*^	65.78 ± 0.12^**^	53.15 ± 0.24^***b^
AO/PI apoptotic IgM cell count (%)	AF2240	2.02 ± 0.90	4.37 ± 0.30^*^	15.34 ± 0.80^**^	17.81 ± 3.70^**^
	IBS002	2.02 ± 0.90	3.29 ± 6.70	10.98 ± 4.40^*^	14.91 ± 3.10^**^
Annexin V positive IgM cell population (%)	AF2240	3.29 ± 1.44	19.72 ± 2.13^*a^	14.35 ± 1.26^**^	34.49 ± 2.50^***a^
	IBS002	3.29 ± 1.44	9.22 ± 2.20^*b^	12.15 ± 1.35^*^	22.81 ± 1.14^**b^

All results represent the mean ± standard deviation (*n* = 3)

Different asterisk signs show significant different between infection time points (*P* ≤ 0.05).

Different alphabets show significant different between different strains of NDV (*P* ≤ 0.05).

**Table 4:** Summary of results

	Infection of NDV AF2240	Infection of NDV IBS002
Cell population changes	• Increment of T lymphocytes and macrophages, depletion of B lymphocytes compared to uninfected control. • Higher increment of macrophages and depletion of B lymphocytes compared of IBS002 infection.	• Increment of T lymphocytes and macrophages, depletion of B lymphocytes compared to uninfected control. • Lower increment of macrophages and depletion of B lymphocytes compared of AF2240 infection.
Oxidative stress	• Increment of NO and MDA content compared to uninfected control. • Higher increment of NO and MDA content compared to IBS002 infection.	• Increment of NO and MDA content compared to uninfected control. • Lower increment of NO and MDA content compared to AF2240 infection.
Expression of pro-inflammatory and anti-inflammatory cykotines/chemokine (CXCLi1, IFNɣ, IL18 and IL10)	• Increment of pro-inflammatory cytokines/chemokines (CXCLi2, IFNɣ, IL18) compared to uninfected control. • Anti-inflammatory IL10 was detected in the bursa at day 3 and 4 post infection but not at day 1 and uninfected control. • Higher increment of pro-inflammatory cytokines/chemokines (CXCLi2, IFNɣ, IL18) compared to IBS002 infection.	• Increment of pro-inflammatory cytokines/chemokines (CXCLi2, IFNɣ, IL18) compared to uninfected control. • Higher anti-inflammatory IL10 was observed in day 3 and day 4 bursa compared to AF2240 infection. • Lower increment of pro-inflammatory cytokines/chemokines (CXCLi2, IFNɣ, IL18) compared to AF2240 infection.
Viral load	• Viral load was detected in in the bursa at day 3 and 4 post infection but not at day 1 and uninfected control. • Lower detected viral load compared to IBS002 infection.	• Viral load was detected in in the bursa at day 3 and 4 post infection but not at day 1 and uninfected control. • Higher detected viral load compared to AF2240 infection.
Apoptosis of IgM+ cells	• Increment of IgM+ apoptotic population over the infection time. • Higher increment of IgM+ cell apoptosis compared to IBS002 infection.	• Increment of IgM+ apoptotic population over the infection time. • Lower increment of IgM+ cell apoptosis compared to AF2240 infection.

## Discussion

Immune reaction to viral invasion is important to combat virus growth and to minimize any damage that can be imposed on the host cells [30]. In this study, the host immune response in the bursa of Fabricius, an organ mainly colonized by B lymphocytes, was assessed upon infection by the avian virus NDV genotype VII IBS002 and genotype VIII AF2240.

The cell population in the chicken bursa was evaluated by flow cytometry and the results showed that in normal chickens, this organ was predominantly occupied by B lymphocytes while the T lymphocyte and macrophage populations were at minimal cell numbers. Different from the cell population changes in chicken spleen upon NDV infection where CD4+ and CD8+ cells declined in their cell numbers when invaded by the two different NDV genotypes [31], our results showed infiltration of both CD4+ and CD8+ T lymphocytes in NDV AF2240 and IBS022 infected chicken bursa. Such a scenario was reported earlier in cases where infections by IBDV and Marek’s disease virus caused infiltration of T cells in the chicken bursa consequently indicating that T cells, especially CD8+ T cells play a role in combating virus in infected cells in the bursa [15, 17, 32]. It was reported by Rasoli et al. [31] that NDV AF2240 and IBS002 caused decrease of the T cell population in chicken spleen. Thus, we postulated that migration of T cells from chicken spleen to the bursa of Fabricius may have taken place during NDV AF2240 and IBS002 infection because in our study, we found that the T cell number was increased in the chicken bursa during infection. Similar to what was reported earlier, participation of T cells in virus pathogenicity was observed by the migration of the T cells from the chicken spleen to the bursa during infection by IBDV [33]. The higher accumulation of intrabursal T cells and macrophages detected in chicken bursa infected by NDV AF2240 than NDV IBS002 was followed by a similar trend of nitric oxide increment in the chicken bursa upon virus infections by both strains of NDV. It was reported that inflammation caused by bacterial and viral infections subsequently caused oxidative damage to both the host cells and the invading microorganisms as result of the action of host defense [34]. A high level of oxidative stress, as indicated by a rise of NO level, was caused by NDV on the organs (Fig. 2). Similarly to what was reported previously, NDV is able to induce the activation of macrophages, which subsequently triggered iNos gene expression and production of NO at the infected site [35]. The production of NO at the infected site may also be related to the cytokines being expressed. Rasoli et al. [31] reported that IBS002 NDV strain induced higher level of IL10 which inhibited the production of IFNγ upon infection. Not only that, the lower macrophage population, which resulted in a lower NO production was also observed in the case of IBS002 infection. Meanwhile, the product of lipid peroxidation known as MDA can cause damage to the cell at the enzyme, protein or DNA levels subsequently leading to cell death [36, 37]. The content of MDA was measured and the results showed significant activity of lipid peroxidation being detected in chicken bursa infected with NDV AF2240 starting on day 3 post infection while NDV IBS002 caused a significant increase of the MDA level after day 1 post infection. However, the subsequent days showed that the infection by NDV AF2240 caused greater activity of lipid peroxidation in chicken bursa compared to NDV IBS002. As lipid peroxidation can be caused by free radicals such as nitric oxide, we postulate that the increment of MDA level in the chicken bursa upon infection with NDV AF2240 and NDV IBS002 is closely related to the increment of the macrophage population which leads to excess production of nitric oxide. This phenomenon suggests that reactive nitrogen species (RNS) and oxygen species (ROS) caused by excess production of NO and lipid peroxidation, respectively play crucial roles in NDV infection as was reported previously [38].

Other than NO and MDA detections, we also evaluated the viral load and cytokine expression in chicken bursa after infection with the two different strains of NDV. The Taqman based real time PCR results showed that no virus was detected from the chicken bursa cell population on the first day of infection as well as in the control chickens. Both NDV AF2240 and IBS002 were detected in the IgM+ cell population starting from day 3 post-infection and the amount of virus increased as the duration of the infection increased. Of the two virus genotypes, NDV AF2240 was reported to have lower amount of virus residing in the cells when compared to infection by NDV IBS002. This result was similar to the recent report by Hu et al. [23] where higher viral load was able to be detected in the bursa of genotype VII NDV infected chicken compared to infections by genotypes IV and IX. However, Hu et al. [23] reported that a higher viral load in the bursa of genotype VII infected chicken was always associated with a higher fold of pro-inflammatory cytokine expression. However, in our case, genotype VIII NDV AF2240 induced higher expressions of the tested proinflammatory cytokines (CXCLi2, IFN-γ and IL-18) (Fig. 4) but with lower viral load (Table 2) in the chicken bursa than in the case of NDV IBS002 infection. Chicken CXCLi2, a proinflammatory chemokine, is expressed by and is chemotactic for monocytes [39]. On the other hand, IL-18 is a pro-inflammatory cytokine secreted by various immune and none-immune cells including monocytes, macrophages and lymphocytes that promotes expression of IFN-γ by T cells [40], which subsequently activates macrophages to produce NO [41, 42]. In our study, higher expressions of CXCLi2, IL-18 and IFN-γ were observed in infection by NDV AF2240, especially on day 3 post infection of the infected bursa correlating with the increased number of CD8^+^ T cells. The drastic increase of IFN-γ in NDV AF2240 infected bursa further activated macrophages to produce NO on days 3 and 4 post infection. However, lower expression levels of CXCLi2, IL-18 and IFN-γ were observed on day 4 post infection particularly in the bursa of NDV AF2240 infected chicken. This may be accounted for by the immune system attempting to restore the drastic elevation of the NO content [43] in the chicken bursa by expressing anti-inflammatory cytokine IL10 [31]. IL10 was not detected in the uninfected and day 1 NDV infected bursa but was detected on day 3 and 4 post NDV infection. Similar to the expression of IL10 in the spleen [31], IL10 expression was higher in the NDV IBS002 infected bursa indicating this cytokine may help to control and lower the level of pro-inflammatory cytokines and NO in the bursa of the NDV IBS002 infected chicken compared to NDV AF2240 infection. Although IL10 expression was associated with a reduction of the proinflammatory cytokines level on day 4, this reaction was not able to relieve the inflammation in the bursa of NDV IBS002 and AF2240 infected chicken.

Infection by both genotypes of NDV was observed to cause decreased viability of IgM^+^ B cells. IgM^+^ B cell was then further enriched from the chicken bursa to further understand the effects of inflammatory stress towards the development of humoral immunity that plays an important role to induce protection via the synthesis of effective antibodies against the virus [44]. As B lymphocytes were observed to be depleted in the bursa upon infection with the virus, the apoptosis assay was carried out. Apoptosis is a controlled cell death mode which is implicated in the pathogenesis of viruses that causes diseases [45]. The hallmarks of apoptosis including cell membrane blebbing, DNA fragmentation and Phosphatidylserine (PS) externalization [46] were evaluated by the AO/PI assay and Annexin V analysis in this study. Our results based on the trypan blue assay showed that NDV AF2240 reduced viability and induced higher apoptotic cell rate in chicken bursa when compared to NDV IBS002. The changes were significantly observed from day 3 post infection. This result was in line with the Annexin V study which also showed that NDV AF2240 induced higher rate of apoptosis in the bursa than NDV IBS002. In the AO/Pi assay, cells showing characteristics of apoptotic cells such as membrane blebbing, chromatin condensation and up taking propidium iodide stain were evaluated. Similar to the Annexin V results, our AO/Pi results demonstrated that the time course infection of NDV AF2240 induced greater magnitude of apoptosis in chicken bursa of Fabricius compared to IBS002. From our results, the cell cycle analysis showed that NDV AF2240 resulted in a higher population of sub G0/G1 compared to IBS002, showing that apoptotic events were higher during NDV AF2240 infection as sub G0/G1 indicated DNA fragmentation, which is also one of the apoptotic cells’ characteristics. The lower degree of B cell depletion in the bursa of NDV IBS002 infected chicken through apoptosis might contribute to the delayed mortality of IBS002 infected chicken compared to NDV AF2240 [31].

Considering that drastic decline in viability and higher apoptotic rate of B cells were detected in chicken bursa infected with NDV AF2240, which is genotype VIII compared to NDV strain IBS002, which belongs to genotype VII with lower virus residue detected in the organ infected by the former, we deduced that oxidative stress induced by the nitric oxide and lipid peroxidation processes which were noticed in the organ during infection might be the primary cause. Furthermore, NO had been reported to cause events of apoptosis in a variety of cells [47, 48], whereas lipid peroxidation could induce apoptosis by depleting ATP production in the cells [49, 50]. Similarly, the significant infiltration of CD8^+^ T cells and macrophages, which led to the elevation of IFN-γ in NDV AF2240 infected chicken bursa caused higher magnitude of organ damage, failure of antibody production and higher mortality in chicken which suggested that the infection of NDV strain AF2240 was far more acute than the infection of NDV strain IBS002 [31]. A summary of the results is listed in Table 4.

## Conclusion

The results of our study demonstrated that the time course infection of NDV AF2240 induced a greater magnitude of apoptosis in chicken bursa IgM^+^ B cells that may further cause impairment of the humoral immunity when compared to IBS002. This might be due to the high level of oxidative stress which led to organ damage in the chicken bursa of Fabricius regardless of the viral load.

## Additional files

Additional file 1: Figure S1. Figure shows (a) Amplification cycles and (b) standard curve for NDV AF2240 NDV (E = 108.7%; R^2^ = 0.984; Slope = 3.13) as well as (c) amplification cycles and (b) standard curve for NDV IBS002 (E = 100.7%; R^2^ = 0.994; Slope = 3.305). (JPEG 109 kb)

## Abbreviations

ANOVA: Analysis of variance
AO/PI: Acridine orange/propidium iodide
BSA: Bovine serum albumin
CD: Cluster of differentiation
cDNA: Complementary DNA
Ct: Cycle threshold
CXCLi2: Chemokine (C-X-C motif) ligand 2
DNA: Deoxyribonucleic acid
EDTA: Ethylenediaminetetraacetic acid
ELD50: Embryo Lethal Dose 50
ELISA: Enzyme-linked immunosorbent assay
FITC: Fluorescein isothiocyanate
GAPDH: Glyceraldehyde 3-phosphate dehydrogenase
IBDV: Infectious bursal disease virus
ICPI: Intracerebral pathogenicity index
IFN- ɣ: Interferon gamma
IgM: Immunoglobulin M
IL10: Interleukin-10
IL18: Interleukin-18
MDA: Malondialdehyde
MDT: Mean death-time
MTT: 3-(4,5-dimethylthiazol-2-yl)-2,5-diphenyl tetrazolium bromide
NDV: Newcastle Disease Virus
NO: Nitric oxide
PBS: Phosphate-buffered saline
PCR: Polymerase chain reaction
PE: Phycoerythrin
PerCp: Peridinin-chlorophyll-protein complex
PS: Phosphatidylserine
RNA: Ribonucleic acid
SPF: Specific-pathogen-free
TBARS: Thiobarbituric acid-reactive substance
