# Supplementary figures and images for: The effects of different velogenic NDV infections on the chicken bursa of Fabricius
Source: BMC Vet Res. 2017 May 31;13:151. doi: 10.1186/s12917-017-1071-y (PMC5452610; doi:10.1186/s12917-017-1071-y)

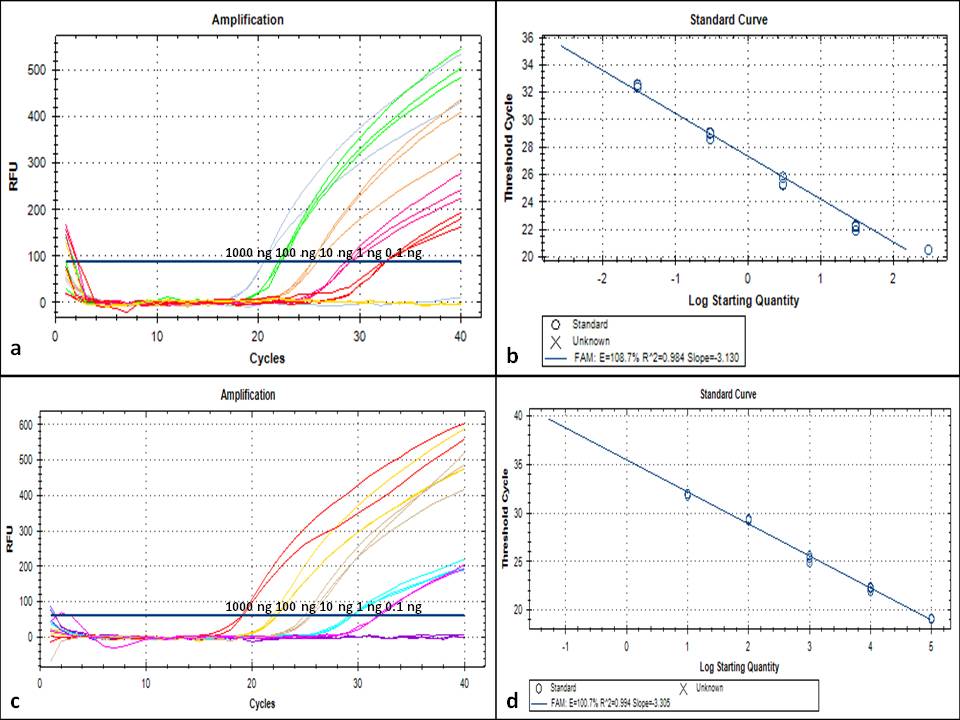

Supplement: Supplementary file 1 — Figure shows (a) Amplification cycles and (b) standard curve for NDV AF2240 NDV (E = 108.7%; R2 = 0.984; Slope = 3.13) as well as (c) amplification cycles and (b) standard curve for NDV IBS002 (E = 100.7%; R2 = 0.994; Slope = 3.305). (JPEG 109 kb) [file 12917_2017_1071_MOESM1_ESM.jpg]
